# Muscle soreness but not neuromuscular fatigue responses following downhill running differ according to the number of exercise bouts

**DOI:** 10.1002/ejsc.12240

**Published:** 2025-02-24

**Authors:** Bastien Bontemps, Julien Louis, Daniel J. Owens, Stella Miríc, Fabrice Vercruyssen, Mathieu Gruet, Robert M. Erskine

**Affiliations:** ^1^ LAMHESS Université Côte d'Azur Nice France; ^2^ School of Sport and Exercise Sciences Liverpool John Moores University Liverpool UK; ^3^ Laboratory Youth‐Physical Activity and Sports‐Health (J‐AP2S) Université de Toulon Toulon France; ^4^ Institute of Sport, Exercise and Health University College London London UK

**Keywords:** eccentric exercise, endurance exercise, exercise‐induced muscle damage, neuromuscular fatigue

## Abstract

Repeated sessions of eccentric‐biased exercise promote strength gains through neuromuscular adaptation. However, it remains unclear whether increasing the number of these sessions can mitigate the extent of neuromuscular fatigue and exercise‐induced muscle damage (EIMD) in response to a standardised eccentric‐biased bout. Twelve healthy untrained adults (five females and seven males; 25.1 ± 4.9 years; and $\dot{V}O_{2\;\max}$: 49.4 ± 6.2 mL kg^−1^ min^−1^) completed two blocks of five downhill running (DR) sessions on a motorised treadmill at a speed equivalent to 60%–65% $\dot{V}O_{2\;\max}$ for 15–30 min. Knee extensor maximal voluntary isometric torque (MVT), electrically evoked measures of neuromuscular fatigue (peripheral and central components), and lower‐limb perceived muscle soreness (PMS) and perceived load (RPE × session duration) were assessed before and immediately after a 15 min standardised DR bout at baseline and after 5 and 10 DR sessions. MVT decreased following a standardised DR bout (*p* < 0.01) similarly at all three time points (−14%, −11% and −9%; *p* > 0.05). The same observations were found for all peripheral and central neuromuscular fatigue indicators after 0, 5 and 10 DR sessions. *Quadriceps* (but not *plantar flexor* or *gluteus*) PMS was lower after 10 DR sessions (8.7 ± 8.5 mm) compared to baseline (29.6 ± 22.2 mm and *p* = 0.01), but no difference was observed after 5 DR sessions (15.4 ± 11.9 mm and *p* = 0.08). Ten repeated sessions of eccentric‐biased exercise led to a reduction in *quadriceps femoris* PMS following a standardised DR bout but neither 5 nor 10 sessions altered the central or peripheral fatigue responses to the same standardised DR bout. These findings suggest distinct physiological adaptations to repeated eccentric‐biased exercise regarding EIMD and neuromuscular fatigue.

## Highlights

- The purpose of this study was to determine whether repeating multiple eccentric‐biased exercise sessions (i.e. downhill running [DR]) can reduce neuromuscular fatigue and exercise‐induced muscle damage responses following a standardised DR bout.
- Neither 5 nor 10 repeated DR bouts were able to significantly reduce either the peripheral or central fatigue response to a standardised DR bout.
- However, after 10 DR sessions, perceived *quadriceps femoris* muscle soreness was reduced.
- These novel data suggest that there are independent physiological mechanisms underpinning the development of muscle damage and neuromuscular fatigue in response to DR.

**Abbreviations**
Db100Hz: potentiated doublets at 100 Hz
Db10Hz: potentiated doublets at 10 Hz
DR: downhill running
EIMD: exercise‐induced muscle damage
EMG: electromyography
ITT: interpolated twitch technique
KE: knee extensors
MVT_ISO_
: maximal voluntary isometric torque
M‐wave: evoked compound action potential response
PMS: perceived muscle soreness
RBE: repeated bout effect
RMS: root mean square
RPE: rating perceived exertion
RTD: rate of torque development
TRIMP: perceived load expressed as session impulse
Tw_pot_
: single twitch
VA: voluntary activation
VL: *vastus* *lateralis*
$\dot{V}O_{2\;\max}$
: maximal oxygen uptake

## 1 INTRODUCTION

Unaccustomed and/or intense eccentric‐biased exercise usually results in transient exercise‐induced muscle damage (EIMD) within hours to days following exercise cessation (Douglas et al., 2017). This results in a decrease in muscle strength, often accompanied by an increase in perceived muscle soreness (PMS), and muscle‐specific proteins and inflammatory markers released into the blood (e.g. creatine kinase and TNF‐*α*) (Ebbeling & Clarkson, 1989; Paulsen, 2012). For example, considerable declines (−14% to −55%) in knee extensor (KE) maximal voluntary isometric torque (MVT_ISO_) have been reported in the literature immediately after an acute bout of downhill running (DR), that is, a whole‐body eccentric‐biased and ecological exercise model (Bontemps et al., 2020). Such a decrease is often associated with an immediate reduction in central drive and an increase in ‘low‐frequency fatigue’, measured through electrically evoked procedures (e.g. the torque–frequency relationship), suggesting the occurrence of both central and peripheral fatigue. It should be emphasised that the latter may impair neuromuscular function within hours to days following eccentric‐biased exercise due to several alterations related to EIMD, for example, ultra‐structural alterations at the sarcomere level and impairments in excitation–contraction coupling (Clarkson & Hubal, 2002; Zhang & Wang, 2020). However, performing a subsequent eccentric‐biased bout separated by several days or weeks is well known to lower the magnitude of EIMD. This physiological–biological adaptive response, usually referred to as the *repeated bout effect* (RBE), reduces the severity of fatigue and/or time to recover from functional (e.g. MVT_ISO_), symptomatic (e.g. PMS) and systemic (e.g. muscle‐specific proteins detected in the blood) responses associated with EIMD (Hyldahl et al., 2017).

Several studies have explored the RBE using the DR model. For example, Byrnes et al. (1985) showed that completing a second DR session 3–6 weeks after the first attenuated the severity of EIMD for up to 48 h after the end of the second task. This is typically associated with a lower increase in circulating intracellular protein concentration, a reduction in lower limb PMS and/or a faster recovery of neuromuscular function (Eston et al., 2000; Khassetarash et al., 2022; McKune et al., 2006; Rowlands et al., 2001). Recently, Khassetarash et al. (2022) reported that the RBE is associated with reduced voluntary activation (VA) deficit following the second DR bout. Although this implies that the RBE may play a major role in neuromuscular fatigue, it is not yet clear whether repeated DR sessions would augment the effectiveness of this protective mechanism. To the best of our knowledge, only Schwane et al. (1987) reported that repeating several DR sessions may limit the increase in lower limb PMS scores following a standardised 45 min DR bout but, more importantly, these authors found that the higher the volume of repeated bouts, the greater the positive effect on PMS. However, in this study, no measures of muscle function were carried out, so it is not clear how repeated DR sessions exert their beneficial effects on functional markers of EIMD and neuromuscular fatigue.

On the other hand, recent evidence suggests that short‐term DR training promotes strength gains through neuromuscular adaptations (Bontemps et al., 2022; Toyomura et al., 2018). Bontemps et al. (2022) reported that just 4 weeks of DR training promoted neural (i.e. increased neural drive) and peripheral (e.g., muscle hypertrophy and increased fascicle length) adaptations, which contributed to the strength gains observed following the training period. Interestingly, Baumert et al. (2021) found that a larger muscle size appears to protect the muscle from EIMD. In addition, a longer fascicle would be associated with lower myofibril elongation during eccentric actions, which could limit the severity of ultrastructural damage (Morgan & Talbot, 2002). In line with this, Balnave and Thompson (1993) reported an attenuated reduction in MVT_ISO_ after a standardised downhill walk following repeated downhill walking sessions over 8 weeks. However, it is still unclear whether repeated DR sessions could play a comparable role in reducing DR‐induced muscle damage and neuromuscular fatigue, which can have significance beyond theoretical insights in specific athletic (e.g. endurance and trail running) or clinical (e.g. rehabilitation) contexts.

Thus, we aimed to examine the change in neuromuscular fatigue and PMS in response to a standardised eccentric‐biased exercise bout following repeated exercise sessions and to determine whether potential protective mechanisms were modulated by the number of sessions (i.e. 5 vs. 10 DR sessions). The DR model was utilised across standardised bouts and repeated sessions to induce eccentric contractions of the *quadriceps femoris*. We hypothesised that repeating five sessions of DR exercise would confer protective mechanisms against neuromuscular fatigue (including both central and peripheral components), and that the greater the number of repeated DR sessions, the greater the protective effects on EIMD and neuromuscular fatigue.

## 2 METHODS

### 2.1 Ethics statement

This study was a part of a larger research project (19/SPS/024), which was granted the ethical approval by Liverpool John Moores University Research Ethics Committee and conformed to the standards regarding the use of human participants in research as outlined in the Sixth Declaration of Helsinki (excluding registration in a database). All participants were informed of the experimental procedures and gave their written informed consent before the study commenced.

### 2.2 Participants

Twelve healthy recreationally active individuals volunteered to take part in the study and completed all sessions (five women and seven men; age: 25.1 ± 4.9 years; height: 1.69 ± 0.08 m; mass: 66.7 ± 13.1 kg; BMI: 23.2 ± 3.3 kg · m^2^; and $\dot{V}O_{2\;\max}$: 49.4 ± 6.2 mL · kg^−1^ min^−1^). Based on the data from Maeo et al. (2015) for the difference in strength loss between bouts (*α*: 0.05 and Power (1 − *β*): 0.8) using the G*Power software (v3.1.9.6, Heinrich‐Heine‐Universität Düsseldorf), 12 participants were necessary for the present study. Participants were free from any medical contraindications and had no history of musculotendinous injuries or plyometric, eccentric and/or heavy resistance training in the six months prior to the study. They had also never performed any DR‐specific conditioning. Further, they were asked to maintain habitual lifestyle habits and physical activity for the duration of the study. None of the female participants was using any form of hormonal contraception or long‐acting reversible contraceptive in the six months prior to the study or during the study itself. In addition, female participants were asked to provide information on the typical length of their menstrual cycle and the number of days since the start of their last menstrual cycle (i.e. the first day of menstruation).

### 2.3 Experimental design

**FIGURE 1:**
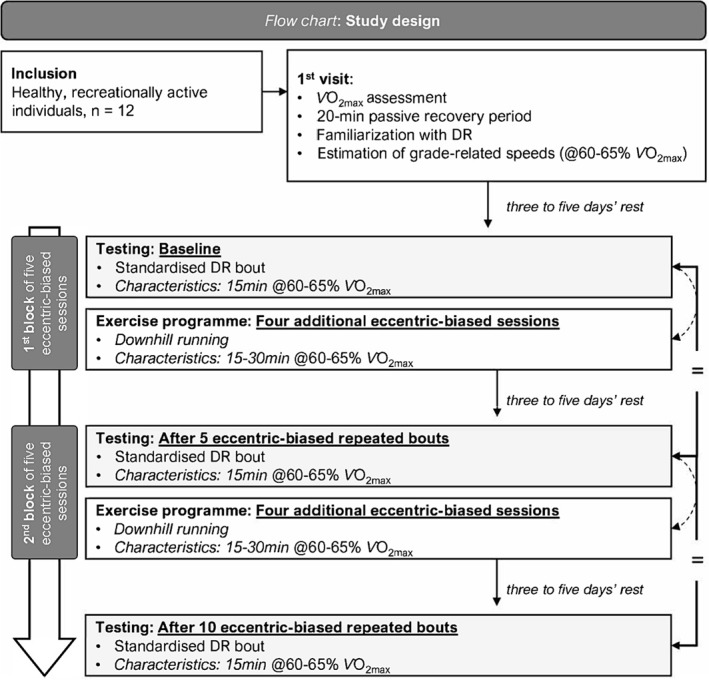
Schematic overview of the study design. DR, downhill running and $\dot{V}O_{2\;\max}$, maximal oxygen uptake.

Participants attended the laboratory on 12 separate occasions (Figure 1). During the first visit, participants performed an incremental running test to volitional exhaustion to determine their maximal oxygen uptake ($\dot{V}O_{2\;\max}$). Following a 20 min passive recovery period, participants were familiarised with DR at three different slopes (−5%, −10% and −15%; i.e. DR_5_, DR_10_ and DR_15_, respectively) at grade‐related speeds associated with 60%–65% $\dot{V}O_{2\;\max}$ for 10–15 min using gas exchange analyses (Oxycon Pro, Carefusion). It allowed for estimating the various grade‐related speeds, which were adjusted if necessary, during the first DR bout. Further, this session enabled the participants to familiarise themselves with all other experimental procedures. The subsequent 11 visits were allocated to DR sessions and/or testing (i.e. visits 2, 7 and 12) sessions. The self‐reported typical menstrual cycle was used to estimate the day of peak luteinising hormone concentration using the regression equation of McIntosh et al. (1980) rounded to the nearest whole day. This allowed the multiple assessment time points (baseline, after 5 bouts and after 10 bouts) to be determined and scheduled as close as possible to the start of the follicular phase (i.e. ± 48 h to the first day of menstruation), thereby reducing any potential effect of fluctuating endogenous oestrogen production on EIMD and neuromuscular fatigue. KE muscle strength, neuromuscular function and lower‐limb PMS scores were evaluated in the right leg before and after a standardised DR bout (see below for details) at baseline (i.e. before starting the first DR session) then after 5 and 10 DR sessions (see below for details). Laboratory conditions remained stable throughout the sessions (temperature: 23.4 ± 1.0°C and relative humidity: 41.7 ± 7.4%).

### 2.4 Exercise programme overview

**FIGURE 2:**
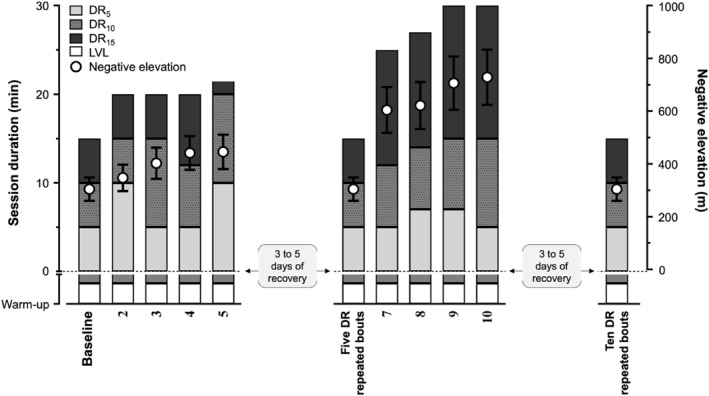
Schematic overview of the supervised downhill running (DR) programme, which consists of two blocks of five DR repeated sessions interspersed by 3–5 days of recovery. Evaluation sessions were conducted at baseline and after 5 and 10 DR repeated sessions. Evaluation sessions were also repeated sessions for the first and second blocks. Each evaluation session included an assessment of neuromuscular function and an examination of perceived muscle soreness scores before and after a 15 min standardised DR bout equivalent to the baseline absolute external load. This figure is not scaled in time.

The supervised programme comprised two blocks of five sessions, interspersed by 3–5 days rest between blocks and/or subsequent evaluations in order to limit the effect of EIMD on neuromuscular function and PMS assessments (Figure 2). Participants were required to conform to the same session schedule (± 1.5 h) for the entire duration of the study. A warm‐up comprising 7 min level running and 3 min DR_10_ at a speed associated with a metabolic intensity of 60%–65% $\dot{V}O_{2\;\max}$ preceded each DR session. DR sessions comprised consecutive treadmill running (HP Cosmos) at DR_5_, DR_10_ and DR_15_ at a speed associated with a metabolic intensity of 60%–65% $\dot{V}O_{2\;\max}$ at each grade (i.e. 8.5 ± 0.9 km · h^−1^, 10.2 ± 1.6 km · h^−1^, 11.7 ± 1.9 km · h^−1^ and 13.0 ± 1.9 km · h^−1^ for the level grade, DR_5_, DR_10_ and DR_15_, respectively). Each DR session was interspersed by 1–2 days rest. Total running time and/or time at steeper slopes was gradually increased throughout the study, regardless of the block, to promote significant stress on the *quadriceps femoris* muscle‐tendon unit.

### 2.5 Standardised DR bout

Participants performed a 15 min standardised DR bout comprising 5 min running at DR_5_, 5 min at DR_10_ and 5 min at DR_15_ consecutively on the treadmill at a speed associated with a metabolic intensity of 60%–65% $\dot{V}O_{2\;\max}$ at each grade (Figure 2). Particular attention was drawn to reducing the time between the standardised eccentric exercise bout and subsequent neuromuscular assessments to limit recovery (< 90 s). PMS scores were measured before and immediately after each standardised DR bout in the *quadriceps femoris*, *plantar flexor* and *gluteus* muscles using a 100 mm visual analogue scale (0 mm corresponding to *no soreness* and 100 mm to *extremely painful*), following five unilateral steps onto a 42 cm highchair seat. In addition, rating of perceived exertion using a 6–20 Borg scale (Borg, 1982) was measured during the last 30 s of exercise at each grade. This measurement captured the exertion specific to each phase of the session enabling the estimation of the participants' perceived load. The final perceived load, expressed as session impulse (TRIMP), was calculated according to the method established by Foster et al. (2001).

### 2.6 Torque measurements

KE isometric voluntary and evoked (potentiated) contractions were assessed using an isokinetic dynamometer (Humac Norm, CSMI), with the hip set at 85° (supine = 180°), the knee at 90° knee flexion and the participant's chest, waist and thigh secured to the chair with inextensible straps. The dynamometer was calibrated, gravity corrected and all settings were individually recorded and re‐used for subsequent visits. Torque measurements were assessed over four MVT_ISO_, each interspersed with 30 s passive recovery and the highest MVT_ISO_ was used for subsequent analyses. Each MVT_ISO_ was followed by evoked and potentiated contractions using femoral nerve stimulations (for details, see below). A warm‐up was carried out prior to the investigations, comprising 13 concentric repetitions (30° · s^−1^) performed with increasing intensity (i.e. ∼10% to perceived maximum effort), followed by two repetitions at ∼80% isometric MVT_ISO_.

### 2.7 Surface electromyography (EMG)

Surface EMG activity was recorded from the right *vastus lateralis* (VL) during voluntary and evoked contractions using surface bipolar electrodes (Ag–AgCl, Blue Sensor N‐00‐S). Following preparation of the skin (shaving, lightly abrading and cleansing with 70% ethanol), two electrodes were attached (20 mm apart) on the skin at a location corresponding to the distal third of the muscle length along the mid‐sagittal plane (according to the SENIAM recommendations; http://www.seniam.org/) and in the direction of the muscle fascicles (identified using ultrasound). The reference electrode was placed on the skin over the right patella. All electrode locations were measured and recorded for relocation during subsequent tests. Surface EMG signals were amplified (100×, differential amplifier 20–450 Hz) and sampled at 2 kHz with the same analogue‐to‐digital converter (MP150 BIOPAC Systems, Inc.) and PC as the torque signal prior to being band‐pass filtered in both directions between 10 and 500 Hz.

### 2.8 Femoral nerve stimulation

KE muscles were stimulated with transcutaneous electrical stimuli delivered to the right femoral nerve via a constant‐current stimulator (DS7A, Digitimer). A 15 mm diameter cathode (Contrôle Graphique) was pressed manually by the investigator onto the femoral triangle, and a 50 mm × 90 mm rectangular anode (Durastick Plus, DJO Global) was attached to the right gluteal fold. The precise location of the cathode was electrically determined using single square wave pulses (200 μs duration) as the position that evoked the greatest single twitches (Tw_pot_) and concomitant evoked compound action potential (M‐wave) response for a particular submaximal electrical current. The femoral nerve was then stimulated in a relaxed state with 75 mA pulses of 200 μs, and this was incrementally increased by 10–25 mA until no further increase in torque was observed (average intensity: 121 ± 30 mA). This amplitude was increased by 30% to ensure supramaximal stimulation during the neuromuscular function assessment.

### 2.9 Peripheral fatigue indicators

Although two single Tw_pot_ were evoked in the resting muscle after the first‐two MVT_ISO_, potentiated doublets at 10 Hz (Db10Hz) and 100 Hz (Db100Hz) were evoked in the resting muscle after the last two MVT_ISO_ (Figure 1). The amplitudes of these potentiated mechanical responses were used as main indicators of peripheral fatigue and may indicate disruption/alterations within the muscle itself. Concomitant peak‐to‐peak maximal M‐waves (M_max_) to single stimuli were measured from the VL muscle. The Db10Hz/Db100Hz ratio was calculated to further investigate low‐frequency fatigue, that is, an indicator of excitation–contraction coupling failure (Martin et al., 2004; Verges et al., 2009). In addition, the peak rate of torque development (RTD_peak_) using the peak slope of the contraction phase was measured during the single Tw_pot_.

### 2.10 Central fatigue indicators

The interpolated twitch technique (ITT) was used to estimate VA capacity. Briefly, ITT was conducted with transcutaneous electrical stimuli (100 Hz doublet) delivered to the right femoral nerve via the constant‐current stimulator, for which one doublet (*d*) was superimposed on the plateau of a MVT_ISO_ and one control doublet (*D*) 2 s after cessation of the MVT_ISO_. These procedures were performed on the last two of the four MVT_ISO_. Voluntary activation (%) was calculated according to the following equation: VA% = 100 × (1 – (*d* × *h*)/*D*), where *d* is the superimposed doublet torque, *h* is the ratio between the torque at stimulation time and peak MVT_ISO_ and *D* is the control doublet torque (Strojnik & Komi, 1998). The VL root mean square (RMS) of the EMG signal over a 300 ms epoch around peak MVT_ISO_ (± 150 ms) was normalised to M_max_ and was used to assess VL activation.

### 2.11 Statistics

All variables are expressed as means ± standard deviation. All data were tested for normality using the Shapiro–Wilk normality test. A two‐factor within‐subjects ANOVA was used to determine the main effects and interaction effect of *pre/post single* (i.e. standardised) *bout* × *repeated DR sessions* for neuromuscular fatigue indicators except for PMS due to its non‐normal distribution (see below). Mixed‐effect models were used, as one data point was missing in one participant. When significant main effects for one‐way ANOVAs or interaction effects for two‐way ANOVAs were found, post hoc pairwise comparisons with Bonferonni adjustments were performed. For PMS, the Friedman test was performed to compare the change between pre/post single bout across time points (i.e. Δ_pre‐post_), as the variable was not normally distributed due to a ceiling effect. When a significant main effect was found in the Friedman test, Wilcoxon post hoc analyses were conducted. The alpha level (*α*) was set at 5% for all statistical analyses. Significance was determined by comparing the *p*‐values from the statistical tests to the set alpha level. Statistical analyses were performed on the GraphPad Prism software (version 8.0; GraphPad Software Inc.). Within time point, Cohen's *d* effect sizes (ES) from *t*‐tests were assessed for all neuromuscular variables to further explore the potential effect of repeated bouts and ES ranked as follows: <0.15 (*negligible*), ≥ 0.15 to < 0.40 (*small*), ≥ 0.40 to < 0.75 (*medium*) and ≥ 0.75 to < 1.1 (*large*) (Hopkins et al., 2009).

## 3 RESULTS

### 3.1 Neuromuscular function

A main effect of *pre/post single bout* but no *pre/post single bout* × *repeated DR sessions* interaction was observed for MVT_ISO_. Post hoc comparisons revealed that MVT_ISO_ was reduced in similar proportions at all time points, that is, −13.9 ± 7.1%, −10.4 ± 7.2% and −9.3 ± 8.8% at baseline and after 5 and 10 DR repeated sessions, respectively (Table 1; *p* < 0.01). Qualitative analyses revealed *medium* (ES = 0.50 and 0.59) effects of repeated bouts on MVT_ISO_ after 5 and 10 DR repeated sessions, respectively, compared to baseline.

**TABLE 1:** Neuromuscular responses to (‘before’ and ‘immediately after’) a standardised 15 min downhill running (DR) bout at baseline, and after 5 and 10 DR repeated sessions.

		Baseline	After 5 DR repeated sessions	After 10 DR repeated sessions	*Pre/Post standardised DR bout × repeated DR sessions*
MVT_ISO_, N · m	Before	219 ± 65	228 ± 87	249 ± 86	*F* (2, 22) = 0.3865
	Immediately after	191 ± 66*	206 ± 81*	226 ± 83*	*p* = 0.68
Tw_pot_, N · m	Before	50.3 ± 18.8	50.6 ± 19.8	58.1 ± 24.5	*F* (2, 22) = 0.6996
	Immediately after	42 ± 17.6*	43.5 ± 17.4*	49.2 ± 20.9*	*p* = 0.51
Db100Hz, N · m	Before	81.3 ± 21.9	83.8 ± 25.8	87.3 ± 26.7	*F* (2, 22) = 0.6688
	Immediately after	74.6 ± 23.3*	77.6 ± 23.7*	83.5 ± 27.4*	*p* = 0.53
Db10Hz, N · m	Before	78.9 ± 32.8	79.7 ± 32.1	91.6 ± 35.6	*F* (2, 22) = 0.1712
	Immediately after	65.5 ± 28.3*	67.8 ± 29.5*	80.1 ± 35.9*	*p* = 0.84
Db10Hz/Db100 Hz	Before	1.02 ± 0.24	0.95 ± 0.2	1.05 ± 0.15	*F* (2, 22) = 1.538
	Immediately after	0.89 ± 0.23*	0.87 ± 0.2*	0.96 ± 0.21*	*p* = 0.24
RFTpeak, N · m · s^−1^	Before	1186 ± 399	1145 ± 351	1349 ± 576	*F* (2, 22) = 0.7062
	Immediately after	1043 ± 378*	1039 ± 308*	1170 ± 435*	*p* = 0.51
Voluntary activation, %	Before	84 ± 8.9	84.2 ± 12.7	86.5 ± 7.3	*F* (2, 22) = 0.3952
	Immediately after	73 ± 13*	77.6 ± 14*	79.6 ± 14.4*	*p* = 0.68
EMG RMS/M‐wave, mV	Before	0.08 ± 0.03	0.07 ± 0.03	0.09 ± 0.04	*F* (2, 16) = 0.8980
	Immediately after	0.08 ± 0.05	0.06 ± 0.02	0.08 ± 0.02	*p* = 0.43

*Note:* Results are presented as mean ± SD.

Abbreviations: Db10Hz, low‐frequency torque; Db100Hz, high‐frequency torque; Db10Hz/Db100Hz, low‐to high‐frequency torque ratio; MVT_ISO_, maximal voluntary isometric torque; Tw_pot_, potentiated single twitch torque; VA, voluntary activation.

*Significant change from before to immediately after the standardised 15 min DR bout (*p* < 0.05).

A main effect of *pre/post single bout* but no *pre/post single bout* × *repeated DR sessions* interaction was observed for all peripheral indicators of neuromuscular fatigue, that is, Tw_pot_, Db100Hz, Db10Hz/Db100Hz and involuntary RTD_peak_ (Table 1). Post hoc comparisons revealed that (i) Tw_pot_ was reduced after a standardised DR bout by −17.5 ± 11.7%, −14.1 ± 8.8% and −15.3 ± 6.1% at baseline and after 5 and 10 DR sessions, respectively (*p* < 0.001); (ii) Db100 Hz reduced after a standardised DR bout by −7.5 ± 8.5%, −7.5 ± 6.6% and −4.5 ± 7.2% at baseline and after 5 and 10 DR repeated sessions, respectively (*p* < 0.01); (iii) Db10Hz/Db100Hz reduced after a standardised DR bout by −12.8 ± 10.4%, −8.5 ± 7.8% and −9.0 ± 12.0% at baseline and after 5 and 10 DR repeated sessions, respectively (*p* < 0.01) and (iv) RTD_peak_ reduced after a standardised DR bout by −16.8 ± 10.1%, −11.3 ± 11.7% and −10.5 ± 7.0% at baseline and after 5 and 10 DR repeated sessions, respectively (*p* < 0.01). Qualitative analyses revealed *small* to *medium* effects of *repeated DR sessions* on Tw_pot_ (ES = 0.24 and 0.34 after 5 and 10 DR repeated sessions, respectively), Db100Hz (ES = 0.41 after 10 DR repeated sessions), Db10Hz/Db100Hz (ES = 0.51 and 0.37 after 5 and 10 DR repeated sessions, respectively) and RTD_peak_ (ES = 0.38 after 5 DR repeated sessions).

A main effect of *pre/post single bout* but no *pre/post single bout* × *repeated DR sessions* interaction was also observed for VA (Table 1). Post hoc comparisons revealed that VA reduced after a standardised DR bout by −13.4 ± 10.9%, −6.5 ± 17.5% and −7.7 ± 17.9% at baseline and after 5 and 10 DR repeated sessions (*p* < 0.01). Qualitative analyses revealed a *medium* effect of *repeated DR sessions* on VA (ES = 0.52 and 0.42 after 5 and 10 DR repeated sessions, respectively). No main effect (*p* = 0.48) or interaction effect (*p* = 0.43) was observed for RMS/M_max_ (Table 1).

### 3.2 Perceived muscle soreness and load

Wilcoxon comparisons revealed that the *quadriceps femoris* PMS score was significantly affected across repeated DR bouts (*χ* ^2^(2) = 7.478 and *p* = 0.024), with no significant changes observed for the *plantar flexors* (*χ* ^2^(2) = 0.977 and *p* = 0.614) or *gluteus* (*χ* ^2^(2) = 0.318 and *p* = 0.853). The *quadriceps femoris* Δ_pre‐post_ PMS score was significantly reduced after 10 (Δ_pre‐post_: 6.7 ± 8.1 mm and *p* = 0.01) but not 5 (Δ_pre‐post_: 8.6 ± 7.0 mm and *p* = 0.08) DR repeated bouts when compared to baseline (Δ_pre‐post_: 23.6 ± 22.2 mm). No significant difference was observed between 5 and 10 DR repeated bouts (*p* = 0.48). The *plantar flexor* PMS score reached 19.1 ± 18.5 mm (Δ_pre‐post_: 11.2 ± 19.0 mm), 14.2 ± 11.2 mm (Δ_pre‐post_: 10.8 ± 10.6 mm) and 8.9 ± 8.4 mm (Δ_pre‐post_: 7.7 ± 9.1 mm) at baseline and after 5 and 10 DR repeated bouts, respectively. Moreover, the *gluteus* PMS score reached 13.0 ± 15.9 mm (Δ_pre‐post_: 11.1 ± 15.9 mm), 6.5 ± 7.4 mm (Δ_pre‐post_: 4.5 ± 7.7 mm) and 6.7 ± 9.8 mm (Δ_pre‐post_: 5.6 ± 10.3 mm) at baseline and after 5 and 10 DR repeated bouts, respectively.

A main effect of *repeated DR sessions* was also observed for TRIMP. Post hoc comparisons revealed that TRIMP was reduced after 10 DR repeated sessions (159 ± 29 AU) when compared to baseline (177 ± 23 AU) (*p* = 0.04) following a normalised 15 min DR bout.

## 4 DISCUSSION

The purpose of the present study was to examine the EIMD and neuromuscular fatigue responses to a standardised eccentric‐biased exercise bout after 5 and 10 repeated bouts and to investigate whether these acute changes were modulated by the number of repeated bouts in healthy untrained individuals. The DR model was utilised across standardised bouts and repeated sessions to induce eccentric‐biased contractions of the quadriceps femoris muscle. Neither 5 nor 10 DR sessions were sufficient to limit the extent of neuromuscular fatigue (including its peripheral and central components) after a 15 min standardised DR bout. However, 10 repeated DR sessions were sufficient to minimise *quadriceps femoris* PMS scores and reduce perceived load after the 15 min standardised DR session. Overall, these results suggest that, although 10 repeated DR sessions appeared to confer the *quadriceps femoris* muscle some protection against EIMD, neither 5 nor 10 DR sessions were able to reduce the associated neuromuscular fatigue response to a standardised DR bout. This suggests that independent physiological mechanisms underpinning the adaptability to DR in terms of the EIMD and neuromuscular fatigue responses to a single DR bout.

It is well known that performing a single, intense, prolonged and/or unfamiliar exercise bout triggers the RBE and, consequently, reduces the severity of neuromuscular fatigue and EIMD in subsequent exercises of similar nature (Clarkson et al., 1992; McHugh et al., 1999; McHugh & Tetro, 2003). Although previous exposure to DR may stimulate the RBE, it was not clear whether a higher number of eccentric exercise bouts could contribute to a greater protection from EIMD particularly following DR. Given that short‐term DR training increases KE MVT_ISO_ through neuromuscular adaptation (e.g. neuromuscular activation, muscle hypertrophy and increased fascicle length) in healthy previously untrained individuals (Bontemps et al., 2022), we hypothesised that such adaptations would confer a greater to resistance to fatigue following standardised DR bouts during a series of repeated DR sessions. However, in the present study, we found that neither 5 nor 10 DR repeated sessions were sufficient to limit the extent of neuromuscular fatigue after a standardised 15 min DR bout. Accordingly, Cadore et al. (2018) did not report a significant protective mechanism on KE MVT_ISO_ after an intense isokinetic eccentric bout on the knee extensors following 6 weeks isokinetic eccentric training (2 sessions/week). Similarly, Michaut et al. (2004) reported no difference in elbow flexor MVT_ISO_ and MVT_CON_ decrements pre‐to‐post eccentric exercise nor reduced neuromuscular fatigue responses (with respect to both peripheral and central components) after 7 weeks eccentric training (three sessions per week) in healthy previously untrained individuals. In the latter study, it should be noted that the decreases in MVT_ECC_ were significantly reduced after the eccentric training programme suggesting that acute neuromuscular responses to exercise may be influenced by the mode of contraction during training. However, as we did not measure MVT_ECC_ before and after the standardised DR bouts, we cannot confirm this hypothesis with regards to DR exercises.

In a systematic review of the literature with meta‐analysis, Lindsay et al. (2020) reported that a third bout of eccentric exercise does not yield significant improvements in isometric strength loss indices or the rate of strength recovery compared to the second bout. This implies that the RBE is primarily effective after the initial strenuous exercise and that additional sessions or more than three bouts may be necessary to further mitigate neuromuscular function alterations following eccentric exercise. Interestingly, Ingalls et al. (2004) reported that microstructural damage was no longer observed after five repeated bouts of murine skeletal muscle lengthening, whereas evidence for impaired excitation–contraction coupling remained. Therefore, it is likely that mechanisms other than microstructural damage were responsible for the torque deficit and associated excitation–contraction coupling failure observed after five repeated muscle lengthening bouts in the study by Ingalls et al. (2004). Excitation–contraction coupling failure is acknowledged as a primary mechanism contributing to exercise‐induced strength loss associated with low‐frequency fatigue (Allen et al., 2008; Warren et al., 2001). This is likely related to the disruption or loss of force‐generating or force‐bearing elements within the muscle following exercise. Consequently, Ingalls et al. (2004) hypothesised that the substantial and enduring decline in muscle strength after repeated bouts of strenuous exercise may be attributed to an inherent muscle protective mechanism during exercise that minimises damage to force‐bearing structures such as excitation–contraction ‘uncoupling’. However, to the best of our knowledge, no study has investigated excitation–contraction ‘uncoupling’, in response to exercise in humans. In the present study, we found evidence for significant and consistent excitation–contraction coupling failure but no *quadriceps* PMS after 5 and 10 DR bouts. Thus, it is possible that the sustained decline in strength in the current study was partly due to excitation–contraction uncoupling. However, since we did not directly measure muscle damage or mechanical changes in the muscle‐tendon unit (e.g. muscle compliance), we are unable to confirm this hypothesis. Moreover, the VA capacity decreased by a similar amount at all three time points following the 15 min standardised DR bout, indicating that decrements in MVT_ISO_ were also mediated by the central component of neuromuscular fatigue.

Prior to the current study, our understanding of the protective effects of more than two repeated DR sessions was limited to the study by Schwane et al. (1983) and the current scientific knowledge on the RBE induced by different modes of strenuous exercises (Clarkson et al., 1992; McHugh & Tetro, 2003; McHugh et al., 1999). Schwane et al. (1983) reported lower *quadriceps femoris* PMS scores after a 45 min DR session following the short duration DR training (1 vs. 2 weeks with five sessions per week) compared with an untrained control group. Interestingly, the authors reported that the higher the number of DR sessions, the greater the protective effects conferred on lower limb PMS scores. In the present study, we also observed that *quadriceps femoris* PMS scores were reduced after 10 but not 5 repeated sessions compared to baseline and the larger DR exercise volume (i.e. 10 vs. 5 sessions) was thus associated with a greater protective effect. However, it was surprising not to observe a larger effect of DR repeated bouts on PMS in the present study as is often reported in RBE studies using DR. The discrepancy between the results from the present study and those from the literature could be explained by the characteristics of the standardised DR sessions (e.g. exercise duration, gradient and running speed). It is possible that a more intense and/or prolonged standardised bout could exacerbate EIMD, and thus further highlight the potential benefits of repeated DR sessions. The *quadriceps femoris* PMS score might be more affected by DR and the subsequent RBE, given the enhanced braking role of the *quadriceps femoris* muscle‐tendon unit during each phase of ground contact in DR (Buczek & Cavanagh, 1990; Devita et al., 2008). Therefore, it should be emphasised that a large variability in responses was observed for lower limb PMS scores, and that it may depend on individual running kinetics and DR kinetics (e.g. specific/manipulated foot strike pattern and preferred stride length and frequency), particularly for those participants with less technical ability and/or DR familiarity.

Although excitation–contraction failure/uncoupling can contribute to the decline in strength following repeated bouts of strenuous exercise, other mechanisms may also occur (Hyldahl et al., 2017). This includes neural adaptations, alterations of mechanical properties, extracellular matrix remodelling and biochemical signalling, all of which work in concert to coordinate protective adaptations. It might partly explain why the participants in the present study showed a reduction in PMS scores, but no significant differences regarding the changes in neuromuscular fatigue after the standardised DR exercise following 5 and 10 repeated sessions. Although intriguing, this finding is consistent with Fernandez‐Gonzalo et al. (2011), who reported a reduction in PMS scores following repeated eccentric bouts but no effect on the change in MVT_ISO_ after a standardised eccentric bout in healthy young females. In contrast, Chen et al. (2009) found a lowered MVT_ISO_ after a fourth controlled eccentric exercise (−34% vs. −22% after the first bout and *p* < 0.05), whereas PMS scores were no longer significant after the second repeated bout. In addition, Maeo et al. (2016) reported significant decreases in MVT_ISO_ and elevated PMS scores after the first bout of downhill walking but not after two, three or four repeated bouts. Although these discrepancies could be multifactorial (e.g. exercise protocols, muscle groups involved, participants' characteristics and inter‐individual variability), one could argue that PMS scores and MVT_ISO_ decrements after exercise may have a distinct aetiology (Damas et al., 2016). Although reductions in MVT_ISO_ are associated with peripheral and central alterations, PMS has been suggested to be associated with structural damage to connective tissue (e.g. perimysium and/or endomysium) and/or the inflammatory processes (Cheung et al., 2003). During the inflammatory response, prostaglandins, bradykinins, and histamine are produced and protein‐rich fluid is released into the muscle due to increased capillary permeability (Smith et al., 1998). The appearance of these inflammatory species and the increase in intramuscular pressure (e.g. due to oedema) induced by the influx of fluid into the muscle can sensitise and stimulate muscle afferents III–IV involved in nociception. Considering that the inflammatory response follows relatively prolonged kinetics, it is plausible that the repeated bouts of DR may have minimised the extent of damage to collagenous structures resulting in a reduction in the immediate elevation of PMS scores but no discernible impact on neuromuscular function.

## 5 LIMITATIONS

We do acknowledge some limitations with our study that could inform future research. Firstly, the present study did not measure the RBE on neuromuscular fatigue and symptomatic responses associated with EIMD after each eccentric‐biased exercise session, thus preventing a more comprehensive understanding of the RBE on the fatigue response to a single exercise bout. However, this methodological configuration could only have been possible with the planning of 10 identical DR sessions in this study in order to control the mechanical stress applied to lower limb muscles. Because the exercise was strenuous for the neuromuscular and musculotendinous system, progressive increases in intensity and duration were therefore preferred. Furthermore, the characteristics of the standardised DR bout and repeated DR sessions (e.g. short duration, variation of slopes between the standardised bout and repeated bouts and the 10–15 min specific familiarisation to DR) could represent a limitation to the study. A more intense and/or prolonged standardised DR bout may have exacerbated EIMD. Nevertheless, this mechanical load still resulted in a relatively moderate level of neuromuscular fatigue in this population as demonstrated by the declines in MVT, VA and all electrical stimulation fatigue indicators immediately after each of the three standardised DR bouts. Furthermore, our study cohort comprised both male and female participants, which may be perceived as a limitation due to potentially increasing variability within the data. However, most studies investigating sex‐dependent responses to eccentric exercise have found no such sex‐differences (Sayers & Clarkson, 2001; Stupka et al., 2001), and as opposed to a single‐sex cohort, we believe that our study cohort is more representative of a young recreationally active population and therefore has a high external validity.

## 6 CONCLUSION

Ten repeated eccentric‐biased exercise sessions led to a reduction in *quadriceps femoris* muscle soreness and perceived load following an isolated standardised exercise bout. However, neither 5 nor 10 DR sessions altered the central or peripheral fatigue responses to the same standardised DR bout. These novel data suggest that the independent physiological mechanisms underpinning the development of muscle damage and neuromuscular fatigue in response to DR.

## AUTHOR CONTRIBUTIONS

All the authors have contributed to the study conception, study design and interpreted results of experiments. All the authors have read and approved the final manuscript.

## CONFLICT OF INTEREST STATEMENT

The authors declare that they have no conflicts of interest. The results of the present study are presented clearly, honestly and without fabrication, falsification or inappropriate data manipulation.
